# CC16 levels correlate with cigarette smoke exposure in bronchial epithelial cells and with lung function decline in smokers

**DOI:** 10.1186/s12890-018-0607-7

**Published:** 2018-03-16

**Authors:** David Chi-Leung Lam, Hoi-Hin Kwok, Wai-Cho Yu, Fanny Wai-San Ko, Cheuk-Yin Tam, Arthur Chun-Wing Lau, Daniel Yee-Tak Fong, Mary Sau-Man Ip

**Affiliations:** 1 Department of Medicine, Li Ka Shing Faculty of Medicine, University of Hong Kong, 21 Sassoon Road, Pokfulam, HK China; 2 Department of Medicine and Geriatrics, Princess Margaret Hospital, Lai King, HK China; 3 Department of Medicine and Therapeutics, the Chinese University of Hong Kong, Shatin, HK China; 4 Department of Medicine and Geriatrics, Tuen Mun Hospital, 23, Tsing Chung Koon Rd, Tuen Mun, HK China; 5 Department of Intensive Care, Pamela Youde Nethersole Hospital, 3, Lok Man Rd, Chai Wan, HK China; 6 School of Nursing, Li Ka Shing Faculty of Medicine, University of Hong Kong, 21 Sassoon Road, Pokfulam, HK China

**Keywords:** Biomarkers, Lung function, Smoking, Spirometry, Forced expiratory volume

## Abstract

**Background:**

Club cell protein-16 (CC16) expression has been associated with smoking-related lung function decline. The study hypothesis was that CC16 expression in both serum and bronchial epithelium is associated with lung function decline in smokers, and exposure to cigarette smoke will lead to reduction in CC16 expression in bronchial epithelial cells.

**Methods:**

In a cohort of community-based male Chinese subjects recruited for lung function test in 2000, we reassessed their lung function ten years later and measured serum levels of CC16. CC16 expression was further assayed in bronchial epithelium from endobronchial biopsies taken from an independent cohort of subjects undergoing autofluorescence bronchoscopy, and tested for correlation between CC16 immunostaining intensity and lung function. In an in-vitro model, bron**c**hial epithelial cells were exposed to cigarette smoke extract (CSE), and the expression levels of CC16 were measured in bron**c**hial epithelial cells before and after exposure to CSE.

**Results:**

There was a significant association between FEV_1_ decline and serum CC16 levels in smokers. Expression of CC16 in bronchial epithelium showed significant correlation with FEV_1_/FVC. Bronchial epithelial cells showed significant decrease in CC16 expression after exposure to CSE, followed by a subsequent rise in CC16 expression upon removal of CSE.

**Conclusions:**

Results of these clinical and laboratory investigations suggested that low serum CC16 was associated with smoking-related decline in lung function, demonstrated the first time in a Chinese cohort. The data also lend support to the putative role of CC16 in protection against smoking-related bronchial epithelial damage.

(Abstract word count: 243)

**US clinical trial registry:**

NCT01185652, first posted 20 August, 2010.

## Background

Tobacco smoking accelerates lung function decline in adults [1, 2] and is the major cause of chronic obstructive pulmonary disease (COPD), although not all smokers will develop COPD. COPD is characterized by airflow obstruction, defined by a reduced ratio of forced expiratory volume in one second to forced vital capacity (FEV_1_/FVC) on spirometry [3]. Some serum biomarkers like interleukin-6 (IL-6), interleukin-8 (IL-8) and monocyte chemoattractant protein-1 (MCP-1) have been found to be associated with airway inflammation in obstructive lung diseases, including COPD [4], asthma and bronchiectasis [5, 6].

Club cell protein-16 (CC16) is a pneumoprotein that is secreted by club cells in the distal airways [7]. Low serum levels of CC16 have been associated with decline in FEV_1_ in patients with COPD in three cohorts [8] and this relationship has also been shown in young smokers who developed COPD later in their lifespan [8]. The serum levels of CC16 were reported to be lower in smokers compared to non-smokers [9]. CC16 is regarded as a marker of peripheral lung injury [10] and its expression is associated with anti-inflammatory action [11]. Exposure to cigarette smoke has been shown to be associated with reduction in the numbers of club cells in bronchial epithelium and serum CC16 concentrations in animal model [12]. Low levels of serum CC16 were associated with decline in FEV_1_ in COPD subjects (ECLIPSE and Lung Health study) [13–16]. Although no significant correlations could be found between serum CC16 levels and COPD severity stratified by FEV_1_ values [15], CC16 expression in the airway was inversely correlated with the severity of airflow limitation in COPD [17]. Experiments with CC16 knockout mouse model suggested that CC16 has anti-inflammatory action and low CC16 expression in murine airway was associated with increased airway inflammation and alveolar loss [12]. Further clinical studies in human subjects will confirm the roles of CC16 in smoking-related lung function decline.

We hypothesized that CC16 expression in the airways and in circulation would be reduced with smoking, resulting in decline in lung function predisposing to airflow obstruction. We addressed this hypothesis through studies conducted on two independent clinical samples, and an in-vitro model of cigarette smoke exposure.

## Methods

### Subject populations

#### A community cohort of male smokers for follow-up of lung function

A community cohort of Chinese subjects had been recruited in year 2000 by random digital dialing for lung function assessment which was done in eight hospitals in Hong Kong, and the data of non-smokers have been used for formulation of local reference values for lung function tests [18, 19]. All the men recruited in year 2000 were contacted and invited to return for this study in 2010. Only men were recruited because we had a small number of female smokers in the recruited sample 10 years ago, and we would not be able to meet the statistical power and sample size requirement for this study if female subjects were included. Lung function (spirometry) assessment and blood for assay of serum biomarker levels were done in this return visit. The study was approved by Institutional Review Board of the University of Hong Kong/Hong Kong Hospital Authority Hong Kong West Cluster (HKHA HKWC 08–428) and the research was carried out in accordance with the Declaration of Helsinki (2008). Informed written consents were obtained, lung function tests were repeated and a 10 ml venous blood sample was obtained from consented subjects. Lung function tests were performed in the lung function laboratories of three hospitals (Queen Mary Hospital, Prince of Wales Hospital and the Princess Margaret Hospitals, Hong Kong) with the Vmax Encore lung function test system (CareFusion, San Diego, CA, USA) according to the American Thoracic Society – European Respiratory Society recommendations [20]. Changes in FEV_1_ and FVC were defined as the difference in the respective values between the latest measurements in 2010 and the corresponding values in 2000, i.e. lung function decline: ΔFEV_1_ (L) = FEV_1(2010)_ – FEV_1(2000)_ and ΔFVC (L) = FVC_(2010)_ – FVC_(2000)._ The annual decline of lung function parameters were calculated with respect to the number of months between the two assessments in years 2000 and 2010 respectively.

#### An independent sample of subjects for correlation of CC16 expression in endoscopic bronchial biopsy with spirometry parameters

An independent sample cohort who presented with sputum cytological atypia was recruited consecutively. The study was approved by Institutional Review Board of the University of Hong Kong/Hong Kong Hospital Authority Hong Kong West Cluster (HK HA HKWC 09–120). All subjects had recent chest imaging which did not show any lesion accountable for the sputum detection of atypical cells. They received lung function tests followed by bronchoscopy with autofluorescence imaging and endobronchial biopsy.

Chronic smokers were asked to stop smoking at least on the day of lung function tests and bronchoscopy. After diagnostic specimens were taken from autofluorescence magenta areas, additional bronchial biopsies were taken from the adjacent green fluorescent areas (green fluorescent areas represent normal autofluorescence from bronchial epithelium).

### Definition of smokers

Non-smokers were subjects who have never smoked in their life-time. Smokers included current smokers and ex-smokers who have been smoking for more than twelve months in the past but have quitted smoking for at least twelve months before recruitment.

### Immortalized human bronchial epithelial cell lines

The origins and characteristics of human bronchial epithelial cell lines were described in supplement. Four immortalized bronchial epithelial cell lines, HBEC-KT 2–5, were used (from John Minna MD, University of Texas Southwestern Medical Center at Dallas, Texas, USA) [21]. Another four bronchial epithelial cell lines were established from endobronchial biopsy of local healthy Chinese subjects [22] with the same methods [21] and these new cell lines were named HKBS62N-KT, HKBS65.2 N-KT, HKBS150N-KT, HKBS160N-KT [22]. All these human bronchial epithelial cell lines were derived from smokers, except HKBS150N-KT which belonged to a non-smoker.

### Enzyme-linked immunosorbent assay (ELISA) for serum biomarker assay

Serum biomarkers were assayed with and ELISA for serum CC16 levels (RD19102220, BioVendor R&D, Asheville, NC, USA). The procedures were all performed according to manufacturers’ protocols.

### CC16 immunohistochemistry

Formalin-fixed paraffin-embedded (FFPE) tissue (4 μm section) of endobronchial biopsies were de-paraffinized and heat-mediated antigen retrieval (95 °C 30mins) was performed with Tris buffer (10 mM Tris, 1 mM EDTA, pH = 9). The endogenous peroxidase activity of the tissue was removed by treatment with hydrogen peroxide (3%, *v*/v). The sections were blocked with protein block reagent (Dako, Carpinteria, CA, USA) for 30 min and then incubated with primary mouse-anti-human CC16 antibody (E11, 1:10000, Santa Cruz Biotechnology, Santa Cruz, CA, USA) or negative control mouse IgG1 (1:10000, Dako) at 4 °C overnight. The specimens were then further incubated with rabbit-anti-mouse secondary antibody (1:400, Dako) at 37 °C for 30 min. Signal amplification was performed with HRP EnVision+ System (Dako), and the coloured product was developed using DAB substrate chromogen system (Dako) for 3 min. The sections were counter-stained with Hematoxylin. The immunohistochemical staining intensity (Grade 0–3) of CC16 expression in bronchial epithelial cells was scored by two independent investigators (DCL and HHK) who were initially blind to the smoking history of the samples. The immunostaining intensity levels of CC16 in bronchial epithelium were scored as 0 (negligible), 1 (weak), 2 (strong intensity) compared with negative serum control. These scores were used for correlation with lung function parameters.

### Cell culture and cigarette smoke treatment

All the eight cell lines were cultured in triplicates in Keratinocyte-serum-free medium (KSFM) supplemented with bovine pituitary extract (50 μg/ml), human recombinant epidermal growth factor (5 ng/ml) (Life Technology, Grand Island, NY, USA) and penicillin and streptomycin (1%, *v*/v) (Life Technology, Grand Island, NY, USA). Cells were kept at 37 °C in 5% CO_2_ incubator. Cells (2 × 10^5^ cells/well) were seeded onto 60 mm dish overnight and were starved in supplement-free medium for another overnight. The same eight cell lines cultured without CSE addition were cultured in triplicates and they were used as controls.

Cigarette smoke extract (CSE) was prepared as described elsewhere [23]. Briefly, aqueous phase CSE was prepared by bubbling two filter-removed commercial cigarettes (12 mg Tar, 1.0 mg nicotine; Marlboro, Philip Morris, Inc., Richmond, VA, USA) into 20 ml PBS. The extract was then filtered through a 0.22 μm syringe filter. The absorbance of the extract at 320 nm was measured and the stock solution was referred as 100% CSE. Cell viability was monitored with Trypan Blue in MTT assays.

### Ribonucleic acid (RNA) extraction and real-time quantitative polymerase chain reaction (PCR) analysis

At time 0, 96 and 192 h, total RNA was extracted by TRIzol reagent according to manufacturer’s protocol. RNA was then reversely transcribed into complementary DNA (cDNA) by the QuantiNova Reverse Transcription Kit (Qiagen, Hilden, Germany). The relative expression of target mRNA for CC16 [24] was quantified by real-time quantitative PCR with QuantiFast SYBR Green Kit (Qiagen) on a StepOnePlus real-time PCR system (Applied Biosystem, Foster City, CA, USA). The primer sequences were listed in Table 1. The relative expression of the target gene was normalized by the level of glyceraldehyde-3-phosphate dehydrogenase (GAPDH) [25] as the reference gene and then calculated by ΔΔC_t_ method.

**Table 1:** Demographics of the 397 male subjects recruited for this study

	Total	Non-smoker		Ever smoker		*P* value
Number (%)	397	168	(42.3)	229	(57.7)	
Age, years (mean ± SE)						NS
2000	47.7 ± 12.5	46.6 ± 12.3		48.5 ± 12.7		
2010	56.5 ± 12.6	55.4 ± 12.3		57.2 ± 12.8		
BMI						NS
2000	24.69 ± 3.26	24.98 ± 3.38		24.48 ± 3.16		
2010	24.69 ± 3.33	24.93 ± 3.38		24.47 ± 3.28		
Smoking amount, Pack-year (mean ± SE)						
2000				17.1 ± 10.9		
2010				24.7 ± 20.3		
Presence of AFL						NS
2000	21 (5.3)	2		19		
2010	65 (17.2)	20		45		
Development of AFL						NS
New AFL	44 (11.1)	18		26		
Stay AFL	21 (5.2)	2		19		
No AFL	332 (83.6)	158		174		
Lung function parameters 2000						NS
FEV1 in liter	3.01 ± 0.65	3.11 ± 0.64		2.94 ± 0.65		
Mean FEV_1_ (% pred)	107.3 ± 2.6	110.3 ± 6.1		105.1 ± 2.3		
FVC in liter	3.80 ± 0.71	3.84 ± 0.72		3.76 ± 0.69		
Mean FVC (% pred)	105.9 ± 4.7	106.9 ± 2.3		105.2 ± 5.5		
FEV1/FVC (%)	79.2 ± 6.9	80.9 ± 5.2		77.8 ± 7.7		
Lung function parameters 2010						NS
FEV1 in liter	2.81 ± 0.66	2.85 ± 0.61		2.79 ± 0.69		
Mean FEV1 (% pred)	100.0 ± 4.3	102.3 ± 1.1		98.3 ± 7.1		
FVC in liter	3.73 ± 0.75	3.74 ± 0.74		3.73 ± 0.77		
Mean FVC (% pred)	104.7 ± 2.3	105.4 ± 4.5		104.1 ± 6.1		
FEV1/FVC (%)	75.2 ± 7.7	76.2 ± 6.6		74.4 ± 8.3		
Serum Biomarker (in 2010)						
Log CC16	0.72 ± 0.22	0.74 ± 0.22		0.69 ± 0.23		0.029*

BMI = body mass index; NS = not significant; AFL = airflow limitation; FEV_1_ = forced expiratory volume in one second; FVC = forced vital capacity. *p < 0.05 for comparison between smokers and non-smokers

### Statistical analysis

Subject demographics were summarized with descriptive statistics. The differences between smokers and non-smokers were examined by student t test, χ^2^ test or Fisher’s exact test where appropriate, using IBM SPSS software version 21. Mean and standard deviation (SD) or standard errors (SE) were used for continuous variables in normal distribution. Pearson correlations were applied to tests for the correlations between clinical parameters of age, smoking status and lung function decline. Partial correlations were applied for correlations of immunohistochemical staining scores with lung function parameters, adjusted for age, gender and smoking status. Multiple linear regression models were built to determine the clinical parameters of age, height, smoking habits, FEV_1_ values and log serum biomarker levels that could independently predict lung function decline. All *p* values were two-sided and *p* < 0.05 was considered statistically significant. For the in vitro experiments, one-way analysis of variance (ANOVA) followed by Tukey’s multiple comparison tests were performed across multiple groups.

## Results

### Patient characteristics

**Fig. 1:**
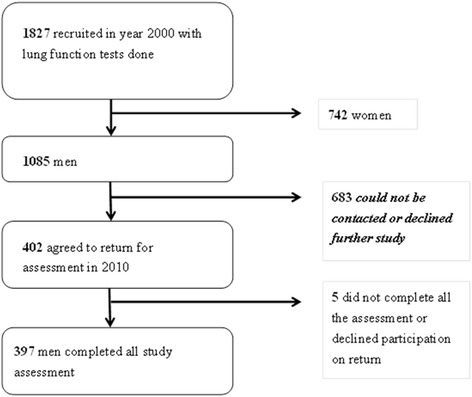
A study flow chart to show the recruitment of subjects

All of the 1085 male consecutive subjects who had lung function tests done in 2000 [18, 19] were contacted again in 2010 (Fig.1). 683 subjects either declined to return for study participation or could not be contacted after six separate attempts (phone calls) at least one week apart at different times. 402 subjects returned after successful phone calls but five of them declined participation upon return, thus 397 subjects completed assessment (Fig. 1). The mean age was 56.5 ± 12.6 years (Table 1). There were 168 non-smokers (42.3%), while 229 (57.7%) were ever-smokers (including 21 subjects who have quitted while the other 208 subjects remained as regular smokers in the last ten years or so). Among ever-smokers, the mean amount of smoking was 24.7 ± 20.3 pack-years. The demographic characteristics of these subjects are summarized in Table 1. These recruited subjects have no significant respiratory symptoms at the time of assessment. Airflow limitation (AFL) (defined as FEV_1_/FVC < 70% on spirometry) [3] was found in 65 (17.2%) of subjects with mean FEV_1_/FVC of 67.5 ± 2.5%, most of which were considered as mild degree of airflow obstruction with mean FEV_1_ (% of predicted) at 98.3% (Table 1).

### Serum levels of CC16 correlated with lung function decline

Serum CC16 levels showed significant correlation with FEV_1_ decline, adjusted for age and smoking amount. The serum level of CC16 was found to correlate with spirometric parameters of FEV_1_ (*r* = − 0.23, *p* = 0.014*), but not FVC (*r* = − 0.108, *p* = 0.054) or FEV_1_/FVC (*r* = 0.212, *p* = 0.158).

When the analyses were separated for smokers and non-smokers, serum CC16 levels showed a significant correlation with FEV_1_ (*r* = − 0.124, *p* = 0.027*) in smokers but not in non-smokers. A significant regression model was found with regression coefficient of 0.091, p = 0.014* suggesting that with every 1 ng/ml decrease in serum CC16 level there could be an additional 56 ml of drop in FEV_1_ over the past 10–15 years, i.e. an average of drop of 3.7–5.6 ml/year in addition to the expected smoking-related decline in FEV_1_ (Table 2).

**Table 2:** Multiple linear regression modeling demonstrated significant prediction of FEV_1_ decline with log serum CC16 levels

	Coefficient	SE	*P* values
Age, years	0.018	0.003	< 0.001*
Smoking amount, pack- years	0.099	0.065	0.126
Log serum CC16 levels	−0.091	0.147	0.014*

*p < 0.05

### CC16 expression in human bronchial epithelium correlated with lung function

**Fig. 2:**
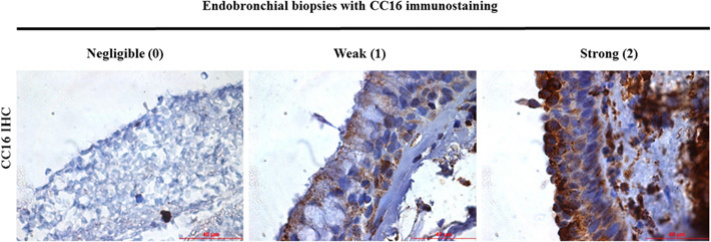
Immunostaining for CC16 expression in endobronchial biopsies showing (A) Negligible staining (scored as 0 with the same intensity in bronchial epithelium) in control and (B) Weak staining (scored as 1) and (C) Strong staining (scored as 2) of CC16 in the bronchial epithelium. Note: Immunostaining were scored with the bronchial epithelial cytoplasmic staining intensity, not the stromal or nuclear staining intensity

Endobronchial biopsies of 34 subjects were evaluated with CC16 immunostaining. The demographic characteristics of these subjects, including smoking status and lung function parameters, are summarized in Table 3. CC16 expression levels (Fig. 2) in their bronchial epithelium showed significant correlation with FEV_1_/FVC (*r* = − 0.689, *p* < 0.001***), adjusted for age, gender and smoking amount. No significant correlation was found between CC16 expression and lung function parameters of FEV_1_ and FVC.

**Table 3:** Demographics of the 34 subjects recruited with endobronchial biopsy for CC16 immunostaining performed

		Non-smokers (*n* = 10)	Ex-smokers (*n* = 11)	Chronic smokers (*n* = 13)
Men*		3 (30%)	11 (100%)	13 (100%)
Age (years)		56.3 ± 14.7	63 ± 9.5	67.4 ± 7.1
FEV_1_ (Liters, % pred)		2.2 ± 0.6 L, 102.3 ± 2.8%	2.4 ± 0.5 L, 97.5 ± 6.5%	2.1 ± 0.3 L, 89.1 ± 5.2%
FVC (Liters, % pred)		2.9 ± 0.6 L, 113.4 ± 4.6%	3.6 ± 0.5 L, 106.0 ± 5.3%	3.4 ± 0.5 L, 92.3 ± 7.2%
FEV_1_/FVC (%)		73.6 ± 11.8	66.6 ± 12.5	62.9 ± 10.8
CC16 intensity				
	0	2 (20%)	3 (27%)	5 (39%)
	1	3 (30%)	4 (36%)	3 (23%)
	2	5 (50%)	4 (36%)	5 (39%)

Significance testing used analysis of variance for continuous variables and chi-square for categorical variables. * *p* < 0.001. FEV_1_ = forced expiratory volume in 1 s. FVC = forced vital capacity. CC16 = Club cell protein-16

### CSE exposure for immortalized bronchial epithelial cells

**Fig. 3:**
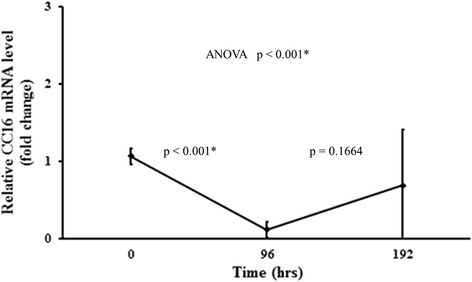
Effects of cigarette smoke extract (CSE) exposure on the expression levels of CC16 mRNA. Eight immortalized normal human bronchial epithelial cell lines were treated with CSE for 96 h after overnight serum starvation. CSE was then removed and washed with PBS for further culture for another 96 h in the absence of CSE. Data were shown as mean +/− standard deviation of eight cell lines and were analyzed by one-way ANOVA followed by Tukey’s multiple comparison tests. The *p* values for comparison of group means at 0 h vs 96 h, and 96 h vs 192 h, are also listed. * *p* < 0.001 vs PBS vehicle control

All the normal bronchial epithelial lines demonstrated consistent initial significant decrease in both CC16 mRNA (*p* < 0.001) and CC16 protein expression (*p* < 0.001) with CSE exposure for 96 h, followed by a non-significant rise in both CC16 mRNA and protein expression levels with removal of CSE (*p* > 0.05)(Fig. 3).

**Fig. 4:**
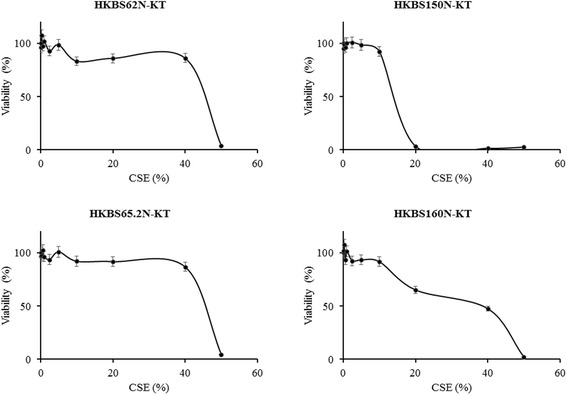
Cell viability monitoring with Trypan Blue and MTT assays for different cell lines during experiments

Cell viability is not affected when the CSE concentration is below 10% (Fig. 4).

## Discussion

In this study we have demonstrated a significant association between CC16 expression and decline in FEV_1_ in smokers but not in non-smokers. In an independent sample of subjects with endobronchial biopsy, there was a significant correlation between CC16 expression in bronchial epithelium and FEV_1_/FVC, i.e. the lower the CC16 expression the lower was the FEV_1_/FVC ratio. This is also the first demonstration of a consistent reduction in both CC16 mRNA and protein expression in immortalized bronchial epithelial cell lines after being exposed to CSE, followed by a subsequent rise of CC16 expression with CSE removal.

The recent report from Guerra et al. demonstrated the relationship between low serum CC16 at baseline and greater lung function decline over the subsequent 8–14 years in three epidemiological cohorts of adults (the population-based Tuscon Epidemiological Study of Airway Obstructive Disease [TESAOD], the European Community Respiratory Health Survey [ECRHS] and the Swiss Cohort Study on Air Pollution and Lung Diseases in Adults [SAPALDIA]) [8]. In that analysis, the three databases designed for different studies were used although the different cohorts shared similar baseline characteristics [8]. Meta-analysis of the data from the three cohorts showed that with decline of CC16 by one standard deviation, there was an associated decline of FEV_1_ by 4.4 ml/year [8]. Baseline CC16 levels were found to be lower in smokers who developed COPD, compared to those smokers who were not found to have COPD later in life, and thus CC16 was suggested to be related to the risk of development of COPD [8]. Our findings that low serum CC16 correlated with greater lung function decline among smokers over the past ten years complement that of previous cohorts. This is the first time such correlation is demonstrated in Chinese. However, such correlation was not evident in non-smokers in our Chinese cohort, while a low CC16 in association with lung function loss in both smokers and non-smokers was reported for Caucasian cohorts [8]. Serum CC16 level reduction could be a more sensitive biomarker for early detection of lung function decline in smokers than in non-smokers. We have only performed one repeat lung function test with a corresponding serum biomarker assessment, and hence we are not able to delineate the pattern of lung function decline over the ten years. Furthermore, we have included only male smokers in our analysis. Given the low female smoking prevalence at 5.6% in the local population, we had a small number of female smokers in the recruited sample 10 years ago, and we would not be able to meet the statistical power and sample size requirement for this study if female subjects were included.

There were previous reports of increase in CC16 mRNA expression in human bronchial epithelial cell lines after exposure to interferon-γ [26], tumor necrosis factor-α [27] and interleukin-10 [28]. These were believed to represent the roles of CC16 in mediating inflammation in airway epithelial cells. This is the first time a direct effect of CSE on bronchial epithelial cells is demonstrated, with reduction in CC16 mRNA expression. The subsequent rise of CC16 mRNA expression in the bronchial epithelial cells upon CSE removal may imply that smoking cessation may well restore the protective role of CC16 against lung function loss.

This study has several unique findings: this is the first report of longitudinal cohort on CC16 and lung function decline in Chinese; the first demonstration of the correlation of CC16 expression in bronchial epithelium and lung function parameter indicative of airflow obstruction; and the first in vitro demonstration of the suppression of CC16 in normal bronchial epithelial cells after cigarette smoke exposure. Although a causal relationship between lower CC16 and worse lung function could not be ascertained from their association as found in the clinical studies, the in vitro study demonstrated that CSE stimulation led to a transient but significant drop in CC16 mRNA expression in bronchial epithelial cells. Further studies into the detailed molecular mechanisms leading to downstream effects of smoking-related transcriptional control of CC16 expression and lung function decline are warranted.

## Conclusions

We demonstrated that serum CC16 levels were associated with lung function decline in this Chinese cohort. The level of CC16 expression in endoscopic biopsies of bronchial epithelium was also found to be correlated with FEV_1_/FVC ratio. To our knowledge, this is the first time that CC16 expression in human bronchial epithelium is shown to correlate with FEV_1_/FVC ratio, a lung function parameter which represents airflow limitation. CSE resulted in significant reduction of CC16 expression in bronchial epithelial cells, with subsequent rise of CC16 expression upon cessation of CSE. This gives further support to the role of CC16 in smoking-related lung function decline and predisposition to chronic airways obstruction, and may imply that CC16 could be a potential target for anti-inflammation in smokers. Further mechanistic studies are warranted to understand the roles of CC16 in translating to smoking-related lung function changes.

## Abbreviations

AFL: Airflow limitation
CC16: Club cell protein-16
COPD: Chronic obstructive pulmonary disease
CSE: Cigarette-smoke extract
ELISA: Enzyme-linked immunosorbent assay
FEV_1_: Forced expiratory volume in 1 second
FFPE: Formalin-fixed paraffin-embedded
FVC: Forced vital capacity
IL: Interleukin
MCP-1: Monocyte chemoattractant protein-1
mRNA: Messenger ribonucleic acid
PCR: Polymerase chain reaction
RNA: Ribonucleic acid
